# Evaluation of antioxidant, anti-hemolytic and anticancer activity of various solvent extracts of *Acacia hydaspica* R. Parker aerial parts

**DOI:** 10.1186/s12906-016-1240-8

**Published:** 2016-07-29

**Authors:** Tayyaba Afsar, Suhail Razak, Muhammad Rashid Khan, Saadia Mawash, Ali Almajwal, Maria Shabir, Ihsan Ul Haq

**Affiliations:** 1 Department of Biochemistry, Faculty of Biological Sciences, Quaid-i-Azam University, Islamabad, Pakistan; 2 Department of Animal Sciences, Faculty of Biological Sciences, Quaid-i-Azam University, Islamabad, Pakistan; 3 Department of Community Health Sciences, College of Applied Medical Sciences, King Saud University, Riyadh, Kingdom of Saudi Arabia; 4 Department of Pharmacy, Faculty of Biological Sciences, Quaid-i-Azam University, Islamabad, Pakistan

**Keywords:** Cytotoxic activity, Antioxidant activity, Phenolic, Flavonoids, Anti-lipid peroxidation

## Abstract

**Background:**

*Acacia hydaspica* R. Parker, family leguminosae, is a medicinally important plant. Different plant parts are used in various ailments in folk medicine. The current study aimed at investigating the in vitro antioxidant, anti-hemolytic and anticancer activity of *A. hydaspica*.

**Methods:**

Antioxidant potential was assessed using DPPH, ABTS and •OH, scavenging of H_2_O_2_, inhibition of lipid peroxidation and β-carotene bleaching inhibition assays. Anti-hemolytic activity was assessed using H_2_O_2_ induced hemolysis of RBCs. Anticancer potential was assessed using MTT assay. Spectrometric methods and HPLC-DAD analysis was performed for phytochemical screening.

**Results:**

EC_50_ values based on reduction of DPPH, ABTS and •OH, scavenging of H_2_O_2_, inhibition of lipid peroxidation and β-carotene bleaching for AHB, AHE and AHM were generally lower manifesting potential antiradical capacities. The fractions also exhibited significant (*P* <0.001) anti-hemolytic potential. Regarding IC_50_ values for anticancer activity against HCC-38 and MDA-MB-361 cancer cell lines; AHB, AHE and AHM exhibited significant (*P* <0.001) cyto-selection indices. Plant extracts showed no cytotoxicity against normal Vero cells (IC_50_ > 250 μg/ml). While significant (*P* <0.001) cytotoxicity was elicited by these extract/fractions against cancer cell lines. AHE was the most effective and IC_50_ was found to be 29.9 ± 0.909 μg/ml (SI = 9.83) and 39.5 ± 0.872 μg/ml (SI = 7.44) against MDA-MB-361 and HCC-38 cancer cells respectively. Higher amounts of TPC and TFC were exhibited by AHE and AHB as compared to other fractions. Gallic acid, catechin and myricetin were identified in AHE whereas gallic acid and catechin were identified in AHB by HPLC.

**Conclusion:**

The presence of bioactive constituents in AHE and AHB might be responsible for antioxidant, anti-hemolytic and anticancer activities.

## Background

Cancer is a prominent reason of death in many developed and developing countries. Although the etiologies of cancer are varied, oxidative stress plays a major role for the pathophysiological developments. Oxidative stress forced by free radicals such as singlet oxygen species, superoxide, hydroxyl and ferrous may cause lipid peroxidation, protein damages, inflammation, autoimmune pathologies, DNA damage, altering cell-signaling pathways and modulating gene expression, induction and promotion of tumor [1, 2]. Synthetic antioxidants, such as butylated hydroxyanisole (BHA) and butylated hydroxytoluene (BHT) are widely used in the food industry because they are effective and less expensive than natural antioxidants. Synthetic antioxidants gained safety concerns and have been restricted due to their DNA damaging and other toxic effects [3]. Complementary and alternative medicine is one of the emerging fields in health care today, especially as supportive medicine in treating diseases like cancer [4]. Plant secondary metabolites such as flavonoids, terpenes, alkaloids, α-tocopherol and carotenoids have received considerable attention in recent years due to their diverse pharmacological properties, including cytotoxic and chemo-preventive effects. The possible health benefits of polyphenol consumption have been suggested to derive from their antioxidant properties [4, 5]. Therefore it is interesting to identify selectivity of plant extracts possessing antioxidant potential against cancer and normal cells. In fact, literature has verified an association between intake of diet rich in fruits and vegetables with a decline in oxidative stress induced disorders [6].

*Acacia hydaspica* R. Parker synonym *A. eburnea*, commonly known as ‘kikar’ family leguminosae is an economically important plant. It is a slender deciduous shrub, 1.2–1.8 m tall, twigs glabrous slightly zigzag; bark smooth, dark grey and 1.2–2.5 cm long stipular spines, seeds 1–8, areole not well marked. The bark and seeds are the source of tannin. Leaves serves as fodder for goats [7, 8]. The bark and seeds are the source of tannins. The plant is locally used as antiseptic. The traditional healers of India use various parts of the plant for the treatment of diarrhea; the leaves and the bark are useful in arresting secretion or bleeding. The pods are helpful in removing catarrhal matter and phlegm from the bronchial tubes. The gum dispels irascibility of the skin and soothes the inflamed membranes of the pharynx, alimentary canal and genito-urinary organs (http://trade.indiamart.com/details.mp?offer=6763150691). Gallic acid, catechin, rutin and caffeic acid have been identified in *A. hydaspica* by HPLC-DAD screening of crude methanol extract, while 7-*O*-galloyl catechin, +catechin and methyl gallate have been isolated from ethyl acetate fraction of *A. hydaspica* (AHE). The *A. hydaspica* possess anti-inflammatory, antipyretic and analgesic potentials [9]. Polyphenolic compounds isolated from *A. hydaspica* induce apoptosis and inhibit cancer cell growth in vitro in breast and prostate cancer cells by modulating various signal transduction pathways [10]. Various species of *Acacia* were investigated for their antioxidant and anticancer potentials in various animal models [11]. The extracts from the bark and heartwood of *Acacia confusa* showed significant antioxidant activity in various antioxidant assays, including free radical and superoxide radical scavenging assays and lipid peroxidation assay as well as hydroxyl radical-induced DNA strand scission assay [12]. *A. mangium* and A. *auriculiformis* heartwood extracts showed excellent quenching ability against DPPH free radicles [13]. The antioxidant activities of bark extract of *Acacia* confusa and some of the isolated constituents from its ethyl acetate (EtOAc) fraction in various in vitro systems together with authentic antioxidant standards revealed that EtOAc fraction showed strong superoxide radical scavenging activity, reducing power, and ferrous ion-chelating ability. Results obtained indicated that the bark extracts from *A. confusa* have a great potential to prevent disease caused by the overproduction of radicals and also it might be used as a potential source of natural antioxidant agent [14]. Heartwood extract of *Acacia catechu* induces apoptosis in human breast cancer cell [15]. However to the best of our knowledge there is no single scientific report demonstrating the antioxidant and cyto-selective anticancer potential of *A. hydaspica*.

In this study qualitative phytochemical screening, total phenolic content (TPC), total flavonoid content (TFC), antioxidant and anti-hemolytic activities of extract/fractions were evaluated. Extracts with potent antioxidant activity were tested against normal (VERO) and cancer cell lines (HCC-38 and MDA-MB-361) in order to evaluate the cyto-selective potential against cancer and normal cells. Furthermore polyphenol constituents in active fractions were determined by HPLC-DAD chromatography using standard reference compounds.

## Methods

### Plant collection

The plant was collected from Kirpa village Islamabad, Pakistan. After identification with the help of relevant literature a voucher specimen was deposited (0642531) at the Herbarium of Pakistan Museum of Natural History, Islamabad.

### Preparation of extract/fractions

The aerial parts (twigs and leaves) of the plant were dried in an aerated but shaded area. Dried material was ground by an electrical grinder to obtain 60 μm powder. The methanol extract was obtained by allowing 3 kg of powder to macerate three times in 95 % methanol (3 × 4000 ml) for five consecutive days. The supernatants were mixed and filtered. Solvent was evaporated by rotary vacuum evaporator (Buchi, R114, Switzerland). The residue was taken to dryness to obtain a viscous mass as the crude methanol extract (AHM). An amount of 12 g of AHM was suspended in water (250 ml) with continuous stirring then successively added (3 × 200 ml) following solvents; *n*-hexane, ethyl acetate, chloroform and *n*-butanol respectively, shake well and each layer was allowed to separate for 3 h in a separating funnel and at last water soluble fraction was obtained (AHA). Each of the fractions obtained were dried using a rotary evaporator. AHM and its five subsequent fractions: AHH, AHE, AHC, AHB and AHA were weighed and expressed in terms of percentage of air dried weight of plant material.

### Chemicals

Ascorbic acid, aluminum chloride, 2,2-azino-bis-(3- ethylbenzothiazoline-6-sulphonic acid) (ABTS), ferric chloride (FeCl_3_), Tween 80, β-carotene, (+)-catechin, gallic acid, rutin, quercitin, potassium persulphate, Folin-Ciocalteu’s reagent, ferrozine, gallic acid, rutin, linoleic acid, 2,2-diphenyl-1-picrylhydrazyl (DPPH), nitro blue tetrazolium (NBT), linoleic acid, phenazine methosulphate (PMS), thiobarbituric acid (TBA) and trichloroacetic acid (TCA) were purchased from Sigma Aldrich (Germany). Deoxyribose, riboflavin, sodium carbonate (Na_2_CO_3_), sodium hydroxide (NaOH), disodium hydrogen phosphate (Na_2_HPO_4_) and hydrogen peroxide (H_2_O_2_) were obtained from Wako Co. (Osaka, Japan). Potassium ferricyanide (K_3_Fe (CN) _6_), triflouroacetic acid, sodium dihydrogen phosphate (NaH_2_PO_4_) and all solvents used were of analytical grade and were purchased from Sigma Aldrich (Germany).

### Preliminary phytochemical screening

The methanol extract and its soluble fractions were subjected to phytochemical analysis by using the methods described previously for the detection of terpenoids, alkaloids [16], saponins, tannins, flavonoids [17], cardiac glycoside [18], reducing sugars [19], pholobatannins, coumarins and anthraquinones [20] by qualitative methods.

### Estimation of total phenolic content (TPC)

The total phenols of AHM and its derived fractions were quantified by previously described spectrophotometric method [21]. TPC was calculated from the calibration curve of gallic acid. Estimation of TPC was recorded in triplicate and expressed as mg of gallic acid equivalent/g of dry sample.

### Estimation of total flavonoid content (TFC)

Aluminium chloride colorimetric technique with slight modifications was used for the estimation of TFC [22]. Quantity of TFC was recorded in triplicate from the calibration curve of rutin and expressed as mg of rutin equivalent/g of dry sample.

### Antioxidant assays

#### Sample preparation

Each sample was dissolved in 95 % methanol at a concentration of 1 mg/ml and diluted to prepare the serialized dilutions (10–500 μg/ml) for various antioxidant assays. Reference standard chemicals were used for comparison in all assays.

### Antioxidant activity assessment assays

#### DPPH radical scavenging activity assay

The DPPH assay was executed following previously established protocol with slight modifications [23]. One hundred milliliter methanol (80 %) was added to 24 mg of DPPH to make the stock solution and the stock was stored at 20 °C till used. For the assay the working solution of DPPH was prepared by diluting the stock with methanol until an absorbance of 0.751 ± 0.02 at 517 nm was achieved. An aliquot of 1 ml DPPH solution was dispensed in 100 μl of the test samples of different concentrations (0–250 μg/ml). The mixture was shaken and placed in the dark for 10 min at room temperature. The absorbance of the mixture was recorded using a UV-1601 spectrophotometer (Shimadzu, Kyoto, Japan) at 517 nm. The decrease in absorbance was correlated with the radical scavenging potential of test samples. The percentage of inhibition was assessed as follow

$$ \%\ \mathrm{DPPH}\ \mathrm{s}\mathrm{cavenging}=\left[\frac{\mathrm{Ad}-\left(\mathrm{A}\mathrm{s}\mathrm{d}-\mathrm{A}\mathrm{s}\mathrm{a}\right)}{\mathrm{Ad}}\right]\times 100. $$

Where Ad is the DPPH solution absorbance, Asd is the absorbance of solution containing test sample and DPPH solution, and Asa is the absorbance sample solution without DPPH. Each sample was analyzed in thrice.

As a standard reference compound ascorbic acid was employed.

#### Superoxide anion radical quenching assay

Quenching potential for superoxide anion was assessed via riboflavin light-NBT system [24]. 1 ml sample solution (25–250 μg/ml) was poured to the solution comprised of 50 mM phosphate buffer (500 μl, pH 7.6), 50 mM riboflavin (300 μl), 20 mM PMS (250 μl) and 0.5 mM NBT (100 μl). Illumination of the solution was done by using a fluorescent lamp for initiating the reaction. The absorbance of samples was recorded at 560 nm after 20 min of illumination. Restraint of superoxide anion liberation was assessed using the following formula:

$$ \mathrm{Inhibitory}\ \mathrm{potentieal}\ \left(\%\right)=\left[\frac{\mathrm{Control}\ \mathrm{abs}-\mathrm{sample}\ \mathrm{abs}}{\mathrm{control}\ \mathrm{abs}}\right]\times 100 $$

Gallic acid was used as a standard compound.

#### Hydroxyl radical quenching activity

Scavenging potential of test samples for the hydroxyl radicals was examined using 2-deoxyribose method [25]. 0.2 M Phosphate buffer saline (PH 7.4) was consumed as a solvent in this test. Sample solution (0–100 μM) was mixed with test mixture containing 2-deoxyribose (2.8 mM), ferrous ammonium sulphate solution (20 mM), EDTA (100 μM). Total volume of test mixture was made up to 1 ml with 0.2 M Phosphate buffer saline (PH 7.4). Ferrous ion solution and EDTA were premixed before adding to the assay mixture. The reaction was initiated by the addition of 100 μl of 20 mM H_2_O_2_ and 100 μl of 2 mM ascorbic acid and incubated at 37 °C for 15 min. Then, thiobarbituric acid solution (1 ml, 1 %, *w/v*) and trichloroacetic acid solution (1 ml, 2 %, *w/v*) were added. The mixture was boiled in water bath for 15 min and cooled in ice, and its absorbance was measured at 532 nm. All experiments involving these samples were triplicated. The scavenging activity were calculated by following formula.

$$ \mathrm{Radical}\ \mathrm{quenching}\ \mathrm{capacity}\ \left(\%\right)=\left[\frac{\mathrm{Control}\ \mathrm{absorbance}-\mathrm{sample}\ \mathrm{absorbance}}{\mathrm{control}\ \mathrm{absorbance}}\right]\times 100 $$

Gallic acid was employed as a reference standard.

#### Hydrogen peroxide radical quenching assay

Hydrogen peroxide solution (200 mM) was prepared in phosphate buffer (50 mM, pH 7.4). 100 μl of test sample (0.1–0.5 mg/ml) mixed with 400 μl of 50 mM phosphate buffer (pH 7.4), then add 600 μl hydrogen peroxide solution and vortex the sample tubes. Note the absorbance of the solution at 230 nm after 10 min against a blank [26].

Hydrogen peroxide scavenging ability is estimated as follow:

$$ \mathrm{Quenching}\ \mathrm{capacity}\ \left(\%\right)=\left[\frac{\mathrm{Control}\ \mathrm{abs}-\mathrm{sample}\ \mathrm{abs}}{\mathrm{Control}\ \mathrm{abs}}\right]\times 100 $$

Ascorbic acid was used as a standard reference.

#### ABTS radical scavenging activity

ABTS test was employed to evaluate the antioxidant prospective of biological fluids, tissues, natural and synthetic complexes. The ABTS^+^ radical cation formation induced by metmyoglobin and hydrogen peroxide is measured by previously establish protocol [27]. ABTS (7 mM) was allowed to react in dark with potassium persulfate (2.45 mM) for 12 h to get a dark shaded ABTS radical cations solution. The ABTS solution used for the assay was prepared by diluting it with methanol (50 %) to achieve an absorbance of around 0.70 at 745 nm. ABTS radical quenching potential was judged by adding 1.0 ml of ABTS working solution in 100 μl of test sample. The drop in absorbance was recorded precisely after one minute, then at 3rd min and last reading recorded at 6th min. The following formula was applied to calculate percentage inhibition:

$$ \mathrm{Quenching}\ \mathrm{ability}\ \left(\%\right)=\left[\frac{\mathrm{Control}\ \mathrm{abs}-\mathrm{sample}\ \mathrm{abs}}{\mathrm{control}\ \mathrm{abs}}\right]\times 100 $$

Ascorbic acid was used as a standard control.

#### Iron chelating power

The extract potency to chelate iron (II) was estimated by previous described procedure [26]. Two hundred microliter each sample (50–250 μg/ml) was mixed with 0.1 ml of FeCl_2_.2H_2_O (2.0 mM) and 0.9 ml of MeOH. The reaction was started by the addition of 0.4 ml of ferrozine (5.0 mM) after 5 min of incubation. The absorbance of the solution was noted at 562 nm after incubation of 10 min. The percent chelating potential (%) was assessed by employing the following equation:

$$ \mathrm{Chelating}\ \mathrm{action}\ \left(\%\right)=\left[\frac{\mathrm{Control}\ \mathrm{abs}-\mathrm{sample}\ \mathrm{abs}}{\mathrm{Control}\ \mathrm{abs}}\right]\times 100 $$

Catechin was used as a reference compound.

#### β- carotene bleaching test

The test was accomplished as per previously described protocol with slight modifications [28]. Twenty-five microliter of linoleic acid and 400 μl of Tween 80 were poured in 500 μg of β-carotene (dissolved in 1 ml of chloroform). Next step was the removal of chloroform under vacuum. After evaporation of chloroform 100 ml of distilled water was added to the residue and shaken well to make β- carotene linoleate suspension. 1 ml of suspension was mixed with test sample (0.1 ml) and the absorbance of the mixture was noted instantaneously against the blank at 470 nm. Next the samples were positioned for 2 h in water bath set at 45 °C. Subsequently the absorbance is recorded again at 470 nm. The antioxidant potency was assessed as percent impediment of oxidation by employing the subsequent equation.

$$ \mathrm{Bleaching}\ \mathrm{inhibition}\ \left(\%\right)=\left[1-\frac{\mathrm{At}0-\mathrm{A}\mathrm{t}120}{\mathrm{Ac}0-\mathrm{A}\mathrm{c}120}\right]\times 100 $$

At0 is the initial absorbance

At120 is the absorbance of solution after 120 min

Catechin and BHT were employed as a standard reference.

#### Anti-lipid peroxidation analysis

This test was performed in accord with scheme described earlier [29]. The extract/fractions were dissolved in methanol to prepare varying concentrations of sample solutions (50–1000 μg/ml). An aliquot of 300 μl of CuCl_2_ solution (0.05 mM) was added to each test tube before adding sample (50 μl) and linoleic acid (100 μl). Mixture was vortexes for 5 s and kept for 20 h for incubation in shaking water bath set at 37 °C. Twenty microliter of BHT (prepared in 10 mM in ethanol) was poured to each test tube to stop the reaction. Solution of TBA was prepared by dissolving 0.67 % TBA in 0.1 M HCl by sonication and momentary heating. Afterward, 3 ml of this freshly prepared solution of TBA (thiobarbituric acid) was added to each sample tube and mixture was vortexed for 5 s. The sample tubes were kept in hot water bath for 10 min. After cooling the sample tubes, the pink aqueous layer was transferred to new test tubes containing 2.5 ml of 100 % *n*-butanol. Mixture was vortexed for 5 s and allowed to settle. Absorbance of pink solution was noticed at 532 nm using spectrophotometer.

Percentage inhibition was measured according to following formula:

$$ \mathrm{Lipid}\ \mathrm{peroxidation}\ \mathrm{impediment}\ \left(\%\right)=\left[\frac{\mathrm{Control}\ \mathrm{absorbance}-\mathrm{sample}\ \mathrm{absorbance}}{\mathrm{control}\ \mathrm{absorbance}}\right]\times 100 $$

BHA was used as a reference standard.

#### Total antioxidant capacity (TAC) (Phosphomolybdate assay)

Phosphomolybdate method was used to determine the antioxidant capacity of compounds [30]. One thousand microliter of assay mixture comprising H_2_SO_4_ (0.6 M), sodium phosphate (0.028 M) and ammonium molybdate (0.004 M) was poured to the sample tubes containing 100 μl of test sample. Incubation of mixture was done for 90 min in hot water bath set at 95 °C. The absorbance of reaction mixture was noted at 765 nm after the samples were cooled.

Ascorbic acid employed as reference standard.

#### Reducing power assay

The protocol of Kumaran was followed for assessing the reducing ability of the extract/fractions [31]. An amount of 0.5 ml of phosphate buffer (0.2 M, pH 6.6) and 0.5 ml of potassium ferricyanide was mixed with 0.5 ml of the extract/fractions (50–250 μg/ml) and incubated at 50 °C for duration of 20 min. 10 % TCA solution (0.5 ml) was added to the reaction mixture in order to stop the reaction. Afterwards, 0.5 ml solution was pipetted out from each reaction mixture tube and permitted to mix with ferric chloride (100 μl) and of distilled water (0.5 ml). Optical density of the chromogen made was note down at 700 nm after incubation of sample for 10 min. Higher absorbance values were proportionated to higher reducing potency.

Values obtained for gallic acid were used as reference standard.

#### Anti-hemolytic activity

Anti-hemolytic potential of extract/fractions was inspected by spectrophotometric procedure as described previously [32]. Five milliliter of blood from a healthy person was collected in EDTA vials and centrifuged for 5 min at 1000 × g. Supernatant was removed and pellet was washed thrice with PBS (0.2 M, pH 7.4) before re-suspending in saline solution (0.5 %). 0.5 ml of the extract/fractions (100–1000 μg/ml in PBS) was dispensed to 1 ml of erythrocyte suspension and incubated at room temperature for 20 min. Next add 0.5 ml of H_2_O_2_ solution made in buffered saline to the reaction mixture for provoking oxidative degradation of the membrane lipids. Subsequently, the samples were centrifuged at 1000 × g for 10 min and the absorbance of supernatant was noted spectrophotometrically at 540 nm. The relative hemolysis was assessed in comparison with the hemolysis in the H_2_O_2_ treated (negative control), which was set as 100 %. For positive control phosphate buffer saline was used. Each set of experiments was performed in triplicate and inhibitory activity of different fractions was calculated and expressed as percent inhibition of hemolysis. Quercetin (100–500 μg/ml) treated in the similar manner was employed as a reference compound. The study protocol was in agreement with Helsinki Declaration. Study approval (Bch#0256) was obtained from the Ethical Review Committee, Quaid-i-Azam University. Islamabad. Informed consent was obtained from persons who participated in the study.

### Cytotoxicity screening

#### Cell lines and cell culture

HCC-38 (CRL-2314™, homosapien, mammary carcinoma epithelial cells, estrogen receptor negative), MDA-MB-361 (HTB-27™, homosapien, mammary gland/breast; derived from metastatic site: brain) and Vero (CCL-81™, normal kidney cells) cell lines were obtained from ATCC (Manassas, VA, USA). MDA-MB-361 and HCC-38 cells were routinely cultured in DMEM/F12, whereas Vero cells were grown in MEM media (Invitrogen), supplemented with 10 % FBS (Invitrogen 16000–044) and 1 % Penicillin/Streptomycin (Invitrogen 15140–122). The cells were incubated at 37 °C in a humidified atmosphere containing 5 % CO_2_ and 95 % oxygen at all times. HCC38 and Vero cells were seeded in 96-well microtiter plates at density 5 × 10^4^ cells/well, whereas MDA361 cells were plated at 1.25 × 10^4^ cells/well and incubated overnight with respective medium described above to obtain a 70 % confluent layer. The monolayer was treated with different concentrations (3.125–25 μg/ml) of the plant extract/fractions and incubated for 48 h at 37 °C. In all experiments a negative control and a positive control were maintained. Negative control contained only growth media while the positive control contained 50 % DMSO.

#### MTT assay

The principle of MTT is based on cellular reduction of soluble yellow MTT tetrazolium salt (3, 4, 5-(dimethylthiazol-2-yl)-2, 5-diphenyl-tetrazolium bromide) to its purple color formazan product by the mitochondrial dehydrogenase in viable cells. MTT assay was used to determine cytotoxicity of *A. hydaspica* crude extract/fractions. After the end of treatment as described above the culture medium was replaced with fresh medium and MTT assay was performed [33]. Absorbance was recorded using a plate reader (Spe 5 M) on 570 nm, with reference wavelength at 690 nm.

#### Estimation of cytotoxicity and IC_50_

Cell cytotoxicity was calculated as a percentage of corresponding control value (non-treated cells) obtained in a minimum of three independent experiments. The half-maximal inhibitory concentration values (IC_50_), defined as the concentration that inhibits 50 % of cell growth, were calculated from concentration-response curves.

Cytotoxicity was measured using following formula:

$$ \mathrm{Cell}\ \mathrm{survival}\ \left(\%\right)=\left[\frac{\mathrm{At}-\mathrm{Ab}}{\mathrm{Ac}-\mathrm{Ab}}\right]\times 100 $$

Where, At = Absorbance value of test compound, Ab = Absorbance value of blank, Ac = Absorbance value of control,

$$ \mathrm{Cell}\ \mathrm{death}\ \left(\%\right)=100-\%\ \mathrm{Cell}\ \mathrm{survival} $$

IC_50_ values were calculated using Graph pad prism 5.

#### HPLC-DAD analysis

##### Preparation of standard for HPLC-DAD

Stock solutions of rutin, kaempherol, myricetin, gallic acid, catechin, caffeic acid and quercetin were prepared in methanol at concentration of 1 mg/ml and diluted with methanol to get 10, 20, 50, 100 and 200 μg/ml for the standard calibration curve. Calibration curves for standard analytes at 10, 20, 50, 100 and 200 μg/ml concentrations were found to be linear.

##### Preparation of samples for HPLC-DAD

Various analytes and plant extract/fractions stock solutions were prepared in methanol, at a concentration of 10–100 μg/ml. Samples were filtered through 0.45 μm membrane filter (Sortolon polymide; Sortorious). All samples were prepared freshly and used immediately for analysis or stored at 4 °C if not analyzed for more than 1 h.

##### Chromatographic condition

Chromatographic analysis was carried out by using HPLC-DAD (Agilent Germany) attached with Sorbex RX-C8 (Agilent USA) analytical column. Briefly, mobile phase A was acetonitrile-methanol-water acetic acid (5: 10: 85: 1) and mobile phase B was acetonitrile methanol- acetic acid (40: 60: 1). A gradient of time 0–20 min for 0 to 50 % B, 20–25 min for 50 to 100 % B, and then isocratic 100 % B till 40 min was used. The flow rate was 1 ml/min and injection volume was 20 μl. Rutin and gallic acid were analyzed at 257 nm, catechin at 279 nm, caffeic acid at 325 nm and quercetin, myricetin, kaempferol were analyzed at 368 nm. Every time column was reconditioned for 10 min before the next analysis. All the samples were assayed in triplicate at ambient temperature. Quantification was carried out by the integration of peak using the external standard method using following formula:

$$ \mathrm{Conc}.\ \mathrm{of}\ \mathrm{S}\mathrm{C}\ \mathrm{in}\ \mathrm{sample}=\left[\frac{\mathrm{Peak}\ \mathrm{area}\ \left(\mathrm{m}\mathrm{A}\mathrm{U}\ast \mathrm{s}\right)\mathrm{of}\ \mathrm{S}\mathrm{C}\ \mathrm{in}\ \mathrm{sample}}{\mathrm{Peak}\ \mathrm{area}\ \left(\mathrm{m}\mathrm{A}\mathrm{U}\ast \mathrm{s}\right)\mathrm{of}\ \mathrm{S}\mathrm{C}}\right]\times \mathrm{Conc}.\ \mathrm{Of}\ \mathrm{S}\mathrm{C} $$

SC is for standard compound

The concentration of standard compound in each fraction was expressed as μg/100 of dry plant powder.

#### Statistical analysis

All assays were performed in triplicates and results are expressed as mean ± SEM. Data of in vitro antioxidant and anticancer assays was analyzed with help of computerized Graph pad prism software to determine the EC_50_ and IC_50_ values. For analyzing the differences among EC_50_ values of different fractions in different antioxidant assays, a Completely Randomized AOV followed by Tukey HSD All-Pairwise Comparison Test was used, alpha set at 0.05 as a level of significance using Statistix 8.1 software. Correlation analysis was performed to determine the correlation between EC_50_ of various antioxidant assays and total phenolic and total flavonoid content.

## Results and discussion

### Extraction yield

*Acacia hydaspica* crude methanol extract (AHM) yield was 15 % of the dry powder, while AHH, AHE, AHC, AHB and AHA yielded 5.27, 27.77, 1.94, 41.66 and 8.05 % respectively, of dry methanol extract. Depending on the nature of solvent used, extraction and fractions yield was recorded differently. AHB and AHE appear to be the best solvents for fractionation giving the maximum yield (Table 1).

**Table 1:** Extraction yield, total phenolic and flavonoid content in *A. hydaspica* methanol extract and its soluble fractions

Extract/fraction	Extraction yield (%)	TPC (mg gallic acid equivalent/g dry sample)	TFC (mg rutin equivalent/g dry sample)
AHM	15.73^e^	87.6 ± 1.23^c^	127 ± 0.52^c^
AHH	5.46^d^	57.7 ± 1.17^e^	71 ± 0.86^a^
AHE	27.77^b^	120.3 ± 1.15^b^	129 ± 1.32^b^
AHC	1.94^f^	36.0 ± 0.95^f^	34.5 ± 1.13^d^
AHB	41.66^a^	129 ± 2.98^a^	139 ± 1.04^a^
AHA	8.05^c^	73.3 ± 1.53^d^	95 ± 0.05^c^

Value are expressed as mean ± SEM (*n* = 3); means with superscript with different letters in the columns are significantly (*p* <0.05) different from each other

### Qualitative phytochemical screening

Diverse assortment of plant secondary metabolites are known to be biologically active compounds and they are responsible for tremendous pharmacological activities for instance antimicrobial, antioxidant, antifungal and anticancer which may benefit in protection against chronic diseases [34]. Table 2 illustrates the qualitative analysis of different classes of phytochemical in *A. hydaspica* methanol extract and its subsequent fractions. Multiple polar and nonpolar chemical constituents were revealed in different extract/fractions of *A. hydaspica*. Tannins, steroids, flavonoid, saponins, cardiac glycosides and terpenoids were found to be present in the all the extract/fractions of *A. hydaspica* plant extract. Alkaloids were detected in all tested extracts except AHC. Coumarins were detected in AHM, AHE and AHA while absent in AHH, AHC and AHB. Phlobatannins did not make their presence in AHC and AHA. Presence of reducing sugars and anthraquinones was confirmed only in AHA and in AHM respectively. Tannins, glycosides, saponins and flavonoids have antioxidant, cytotoxic, antitumour hypoglycemic and anti-inflammatory activities [35–37]. Terpenoids, and steroids shows analgesic properties and central nervous system (CNS) activities [38]. Various studies confirm that flavonoid groups exhibit high potential biological activities such as antioxidant, anti-inflammatory and anti-allergic reactions [39].

**Table 2:** Phytochemical constituents of *A. hydaspica* methanol extract and its fractions

Compound Class	Samples					
	AHM	AHH	AHC	AHE	AHB	AHA
Tannins	+	+	+	+	+	+
Steroids	+	+	+	+	+	+
Saponins	+	+	+	+	+	+
Alkaloids	+	+	−	+	+	+
Flavonoids	+	+	+	+	+	+
Coumarins	+	−	−	+	−	+
Terpenoids	+	+	+	+	+	+
Pholobatanins	+	+	−	+	+	−
Reducing sugars	−	−	−	−	−	+
Anthraquinones	+	−	−	−	−	−
Cardiac Glycosides	+	+	+	+	+	+

Negative sign (−) indicate absence, positive sign (+) indicate presence. *AHM Acacia hydaspica* methanol extract, *AHH Acacia hydaspica* n-hexane fraction, *AHC Acacia hydaspica* chloroform fractions, *AHE Acacia hydaspica* ethyl acetate fraction, *AHB Acacia hydaspica* n-butanol fraction, *AHA Acacia hydaspica* soluble aqueous fraction

### Total phenolic and flavonoid content

The profile of TPC and TFC in AHM and its derived fractions was determined from the standard calibration curve of gallic acid (R^2^ = 0.92) and rutin (R^2^ = 0.91) respectively. TPC varied widely, ranging from 36.0 ± 0.95 to 139 ± 1.04 mg gallic acid equivalent/g dry sample in the extract/fractions of *A. hydaspica*. However TFC varied from 34.5 ± 1.13 to 129.0 ± 2.98 mg rutin equivalent/g of dry sample. AHB showed the highest concentration (*P* <0.05) of TPC and TFC, followed by AHE > AHM > AHA > AHH > AHC (Table 2). So it is evident from the present data that AHB and AHE are the best solvents for fractioning polyphenol constituents, due to their polarity index and the best solubility for the type of metabolites in *A. hydaspica*. The results obtained in this study were in line to those of Sultana et al. [40], where the highest concentration of TPC had been determined in the bark of *Acacia nilotica*.

### Antioxidant activity assessment

Various reaction mechanisms are usually involved in measuring the antioxidant capacity of a complex samples and there is no single broad-spectrum system which can give an inclusive, precise and quantitative prediction of antioxidant efficacy and antiradical efficiency [41]. Hence more than one technique is suggested to value the antioxidant potential [42]. The EC_50_ values of various antioxidants assays were given in Table 3.

**Table 3:** EC50 values of different antioxidant activities of extract and derived fractions of *A. hydaspica*

EC50 values									
Sample	DPPH radical	Superoxide radical	Hydroxyl radical	Hydrogen peroxide radical	ABTS radical	Iron chelating power	β-carotene bleaching inhibition	Total antioxidant index at 250 μg/ml	Lipid peroxidation inhibition
AHM	19.5 ± 0.43^a^	43.0 ± 0.37^b^	39.0 ± 0.44^a^	40.3 ± 0.26^a^	193 ± 0.26^d^	41.7 ± 0.34^a^	86.4 ± 0.62^d^	1.6 ± 0.69^b^	156.1 ± 0.76^d^
AHH	46.5 ± 0.55^b^	59.3 ± 0.36^c^	42.3 ± 0.29^b^	80.6 ± 0.43^c^	401 ± 0.23^e^	91.7 ± 0.17^b^	173.5 ± 0.85^d^	1.2 ± 0.35^c^	562.3 ± 0.55^g^
AHE	18.0 ± 0.45^a^	38.3 ± 0.40^a^	35.7 ± 0.40^a^	37.2 ± 0.36^a^	173 ± 0.11^c^	40.3 ± 0.21^a^	48.4 ± 0.55^b^	1.7 ± 0.61^b^	61.3 ± 0.52^c^
AHC	65.0 ± 0.36^c^	90.8 ± 0.55^d^	63.0 ± 0.09^c^	115.6 ± 1.51^d^	600 ± 0.26^e^	130.1 ± 0.17^d^	260.6 ± 1.05^f^	0.7 ± 0.73^e^	467.3 ± 0.60^f^
AHB	16.7 ± 0.43^a^	49.0 ± 0.41^b^	33.0 ± 0.16^a^	42.0 ± 0.11^a^	98.0 ± 0.12^a^	39.0 ± 0.26^a^	66.2 ± 0.72^c^	1.7 ± 0.41^a^	50.0 ± 0.61^b^
AHA	39.5 ± 0.51^b^	46.3 ± 0.52^b^	47.0 ± 0.12^b^	68.1 ± 0.36^b^	478 ± 0.12^e^	103.0 ± 0.12^c^	131.6 ± 1.15^e^	1.3 ± 0.14^e^	401.1 ± 0.12^e^
AA	15.0 ± 0.41^a^	-	-	35.0 ± 0.21^a^	61.0 ± 0.21^b^	-	-	1.8 ± 0.24^b^	-
GA	-	32.3 ± 0.28^a^	29.0 ± 0.16^a^	-	-	-	-	-	43.5 ± 0.86^a^
C	-	-	-	-	-	37.7 ± 0.3^a^	40.4 ± 1.01^a^	-	-
BHA	-	-	-	-	-	-		-	59.3 ± 0.98^c^
BHT	-	-	-	-	-	-	50.11 ± 0.59^b^	-	

Values are expressed as Mean ± SEM (*n* = 3). Means with superscripts with different letters in the column are significantly (*P* <0.05) different from each other. *-* indicate not determined, *AHM A. hydaspica* methanol extract, *AHH n*-hexane fraction, *AHE* ethyl acetate fraction, *AHC* chloroform fraction, *AHB n*-butanol fraction, *AHA* aqueous fraction, *AA* Ascorbic acid, *GA* Gallic acid, *C* Catechin, *BHA* butylated hydroxy anisole, *BHT* butylated hydroxy toluene. (Completely Randomized AOV followed by Tukey HSD All-Pairwise Compariso Test)

### DPPH radical scavenging activity

DPPH free radical quenching test is one among the most widely employed procedures to assess antioxidant potency of plant and biological samples [26]. In the current testing; AHM, AHB and AHE depict appealingly greater DPPH quenching efficacy as compared to all other tested fractions and displayed lower EC_50_ values, which were non-significantly different from one another. The EC_50_ values in DPPH assay range from 16.7 ± 0.43–65 ± 0.36 μg/ml. AHB and AHE showed the lowest EC_50_ values (16.7 ± 0.43 and 18 ± 0.45 μg/ml), which were comparable to EC_50_ of reference compound (Ascorbic acid). DPPH radical scavenging activity of *A. hydaspica* extract and its various fractions showed good correlation with TPC (R2 = 0.9879) and TFC (R2 = 0.8477). Results of present investigation imply that *A. hydaspica* contain phyto-constituents that are proficient of donating hydrogen to a free radical in order to rescue the potential impairment. Most specifically, phenolic and flavonoid exhibit antioxidant activity due to the hydroxyl group attach to the aromatic ring which is capable donating electron and stabilizing the free radicals. The research conducted by Sultana et al. [40] in *A. nilotica* and Singh et al. [43] on *Acacia auriculiformis* revealed similar results.

### Superoxide radical scavenging activity

Although superoxide anion is a weak oxidant, but it lead to the generation of powerful and hazardous hydroxyl radicals as well as singlet oxygen, both of which contribute to oxidative stress. Therefore, it is very important to study the scavenging of superoxide anion [44]. The EC_50_ values in superoxide scavenging activities were in the order of AHE < AHM < AHB < AHA < AHH < AHC. When compared to ascorbic acid, the superoxide scavenging activity of the AHE was found to be statistically (*P* >0.05) similar. The potent electron scavenging ability of the methanol extract and its various fractions might be due its bioactive phytoconstituents like that are able to minimize the oxidation of biological macromolecules [26, 37]. A significant correlation was detected with TPC (R^2^ = 0.7909, *P* <0.05) while nonsignificant correlation (*R*^2^ = 0.641, *P* >0.05) was observed with TFC. This strong superoxide radical neutralizing capacity of *A. hydaspica* might be functional therapeutically against oxidative stress induced ailments.

### Hydroxyl radical scavenging activity

The evidence of OH radical scavenging activity by *A. hydaspica* extract and its fractions was determined by measuring the inhibition of 2-deoxyribose degradation by the free radicals generated during Fenton reaction. Crude methanol extract and derived fractions markedly scavenged OH radicals and prevented the degradation of 2-deoxyribose. A dose dependent mode was observed for hydroxyl radical scavenging activity. The lowest EC_50_ values were shown by AHB (36.0 ± 0.16 μg/ml) followed by AHE and AHM (37.7 ± 0.40 and 40.0 ± 0.44 μg/ml respectively). However EC_50_ values were significantly different from standard gallic acid. A significant correlation was observed with TPC (*R*2 = 0.844, *P* <0.01) and TFC (R2 = 0.776, *P* <0.05). The strong antioxidant activity of AHB and AHE might be utilized as a source of natural antioxidant in oxidative stress for minimizing the detrimental effects of hydroxyl radical in the body. A high scavenging activity of AHE for OH radical was reported in one of previous studies done in our lab.

### Hydrogen peroxide radical scavenging activity

In the body, H_2_O_2_ is rapidly decomposed into oxygen and water and this may produce hydroxyl radicals (•OH) that can initiate lipid peroxidation and cause DNA damage [6]. Therefore, the ability of plant extracts to scavenge hydrogen peroxide was also determined in order to get the idea that whether samples have same pattern of activity as OH radical reducing ability. Methanol extract/fractions of *A. hydaspica* possess significant ability to quench the hydrogen peroxide radicals, demonstrating the antioxidant potential of the plant. AHE proved to be efficient fraction against hydrogen peroxide (EC_50_ = 37.2 ± 0.36 μg/ml). EC_50_ values showed significant correlation with both TPC (*R* 2 = 0.844, *P* <0.01) and TFC (R2 = 0.776, *P* <0.05), attributing the activity to the occurrence of polyphenolic compounds that give electrons to hydrogen peroxide, thus neutralizing it into water. These findings are in line with the previous study.

### ABTS radical scavenging assay

In this assay, the reaction of ABTS with potassium persulfate in the existence of hydrogen-donating antioxidants outcomes in the formation of ABTS^+^ blue/green chromophores, this reduction reaction is noted spectrophotometrically at an absorbance of 745 nm. This scheme of determination of antioxidant action is equally pertinent to hydrophilic and lipophilic classes of antioxidants like; flavonoids, hydroxycinnamates, carotenoids and antioxidants in the plasma [45]. The result obtained indicated that AHM and its derived fractions scavenge the ABTS radicals in a dose dependent pattern. Among the extract/fractions lowest EC_50_ values for ABTS radical scavenging were determined for AHB (98.0 ± 0.1 μg/ml) while highest EC_50_ values were recorded for AHC (>500 ± 0.26 μg/ml) as shown in Table 1. However EC_50_ values of AHB were significantly lower than ascorbic acid (61 ± 0.2 μg/ml). The ABTS scavenging activity of the present study suggests that the phyto-constituents within the extract and various fractions of *A. hydaspica* might donate electron/hydrogen while minimizing the oxidative stress. Furthermore correlation analysis indicated significant correlation between ABTS radical scavenging activity and TPC (R^2^ = 0.881, *P* <0.001) as well as TFC (R^2^ = 0.857, *P* <0.001). The results are in line with the study of khan et al. [46].

### Chelating activity on Fe2+

An imperative mechanism of antioxidant activity is the ability to chelate/counteract transition metals, which have the ability to demolish hydro peroxides and Fenton-type reactions. Therefore it was considered important to screen the iron (II) chelating ability of extract/fractions. The sequence for chelating power was AHB ~ AHE ≥ AHM > AHH > AHA > AHC. The EC_50_ values of AHB, AHE and AHM were close to the EC_50_ value of standard catechin. Correlation analysis suggested that the iron (II) chelating possessions of *A. hydaspica* may be accredited to its endogenous chelating agents like polyphenolic compounds [47]. As some phenolic compounds have properly oriented functional groups, which can chelate metal ions to protect against oxidative damage [48]. Chelating activity was correlated well with the phenolic (R2 = 0.8971, *P* <0.001) and flavonoid content (R2 = 0.8177, *p* <0.05) Our results are in good agreement with previous report where ethyl acetate fraction possessing polyphenolic constituents of *A. auriculiformis* showed highest metal chelating power [43].

### β-carotene bleaching inhibition

In this test, antioxidant competence was evaluated by quantifying the inhibition of the conjugated diene-hydroperoxides and the volatile organic compounds creation as an outcome of linoleic acid oxidation. Hence, the antioxidant occurrence can impede the magnitude of β-carotene bleaching by counterbalancing the linoleate and other free radicals formed in the process. The color of reaction solution was retained for a long time in the presence of an antioxidant compound while a rapid decrease in absorbance was noticed in the absence of antioxidant [49]. The antioxidant activity of *A. hydaspica* extract/fractions with regard to the β-carotene bleaching method could be ranked as AHE > AHB > AHM > AHA > AHH > AHC. The EC_50_ values of AHE (48.4 ± 0.55 μg/ml) were significantly different yet comparable with standard catechin (EC_50_ = 40.4 ± 1.01 μg/ml). However AHE showed better efficacy as compared to standard BHT. The differential efficacy of *A. hydaspica* extract/fractions to inhibit oxidation of linoleic acid emulsion is an indication of the complexity of the extract/fractions as well as potential interaction between the extract and emulsion components. Correlation analysis indicate significant correlation with phenolic (*R*2 = 0.9670, *P* <0.0001) and flavonoid (*R*2 = 0.8831, *P* <0.001) content. The correlation of phenolic and flavonoids with β-carotene bleaching inhibition potential was reported by other researchers as well [26, 50].

### Anti-lipid peroxidation potential

Lipid peroxidation is involved in a number of pathological conditions so evaluation of antioxidant potential of natural and synthetic compounds requires an assay in lipid system too. The EC_50_ values in lipid peroxidation inhibition were in the order of AHB < AHE < AHM < AHA < AHC < AHH. EC_50_ values showed by AHB (50 ± 0.61 μg/ml) were significantly lower as compared to BHA values (EC_50_ = 59.3 ± 0.98 μg/ml). In this study the in vitro ability of plant extract/fractions to prevent the production of TBARS depicts the potential of samples to inhibit oxidation in lipid system. Significant correlation was observed with TPC (*R*^2^ = 0.779, *P* <0.05) and TFC (*R*^2^ = 0.836, *P* <0.05). Phenolic compounds are very important plant constituents because they exhibit antioxidant activities by inactivating lipid free radicals, or by preventing the decomposition of hydro-peroxides into free radicals. Results of lipid peroxidation were in line with the previous study of Singh et al. [43].

### Total antioxidant capacity assay

The total antioxidant capacity of the extract/fractions was measured by phosphomolybdenum method based on the reduction of molybnedum (VI) to molybnedum (V) by the antioxidant action of extract and the subsequent formation of a green phosphate Mo (V) complex at acid pH of the medium with maximum absorbance at 695 nm [51]. Total antioxidant capacity of the extract/fractions recorded at the highest dose of 250 μg/ml was in order of AHB (1.7 ± 0.015) ~ AHE (1.7 ± 0.08) > AHM (1.5 ± 0.016) > AHA (1.3 ± 0.05) > AHH (1.2 ± 0.02) > AHC (0.738 ± 0.012). The current analysis reveals that AHB and AHE displayed the uppermost antioxidant capacity. Latest researches proved that flavonoids and related polyphenols contributes substantially to the phosphomolybdate quenching capability of medicinal plants [46, 52]. AHB and AHE exhibited the highest antioxidant index comparable with ascorbic acid. Phosphomolybdenum assay in general detects antioxidants such as carotenoids, ascorbic acid, $\alpha$-tocopherol, and some phenolic, cysteine, and aromatic amines due to hydrogen and electron donating ability. The antioxidant capacities of the extract/fractions have a strong relationship with the solvent employed, mainly due to the different antioxidant potential of compounds with different polarities. Phytochemical analysis reveals the presence of various boiactive phytochemicals that might be attributed to the antioxidant capacity of *A. hydaspica*. Our result correlate well with the research of Tung at al. reporting gallic acid, catechin, myricetin along with other polyphenols in *A. confusa* leaves extract were responsible for the significant antioxidant potential [53].

### Reducing power

**Fig. 1:**
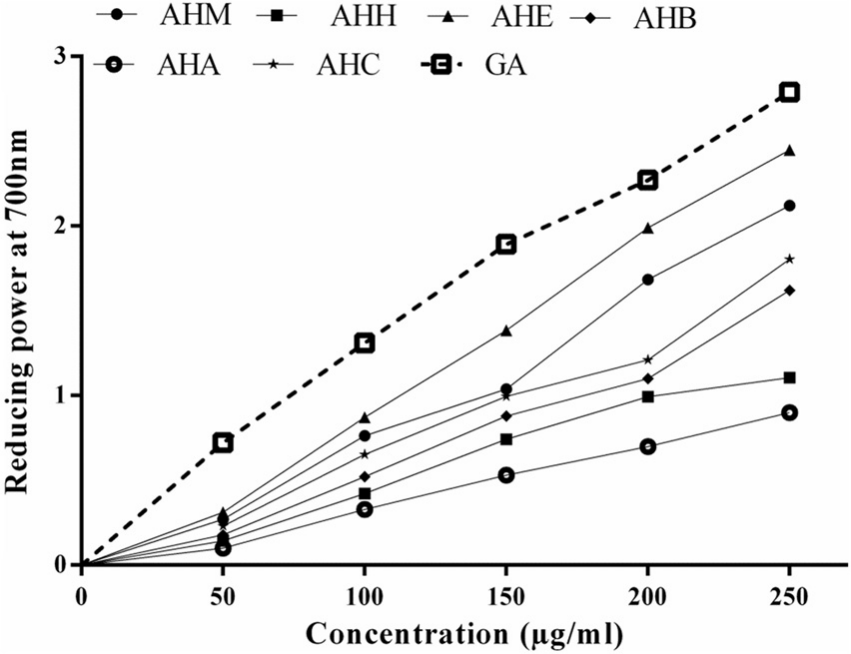
Dose dependent reducing power activity of extract and fractions of *A. hydaspica*. Values are expressed as mean ± SEM (*n* = 3). *GA* Gallic acid used as reference

Samples owning more reducing efficacy displayed high absorbance values. From the literature, it’s evident that reducing power is attributed to the existence of antioxidants that contributes hydrogens atoms to the free radicals [54]. It was observed that reducing power of the extract/fractions was increased in a dose dependent manner. The reducing power of the extract and various fractions was in an order of AHE > AHB > AHM > AHH > AHC > AHA. The AHE and AHM exhibited an excellent reducing power of 2.5 ± 0.024 and 2.3 ± 0.09 at a concentration of 250 μg/ml (Fig. 1). AHE at 250 μg/ml showed good reducing power that was comparable to standard Gallic acid. The significant reducing potential of AHE might be credited to the elevated amount of polyphenols that may function as indicator of its potential antioxidant action. These results are in agreement with the previous study where ethyl acetate extract of *A. auriculiformis* bark exhibit significant reducing potential [43].

### Anti-hemolytic activity

The most plentiful cells in the human body are found to be the erythrocytes, which own copious biological and morphological characteristics, hence they have been widely exploited in drug transport. The polyunsaturated fatty acids (PUFA) and hemoglobin molecules which are redox active oxygen transport molecules and potent promoters of activated oxygen species mainly target the erythrocytes. Oxidative mutilation to the erythrocyte membrane lipids and proteins may be responsible for hemolysis accompanying with several factors viz. hemoglobinopathies, oxidative drugs, excess of transition metals, various radiation, and deficiencies in erythrocyte antioxidant coordination [55, 56]. The magnitude of hemolysis was appeared to be much more overwhelming, when red blood cells were exposed to any toxicant like hydrogen peroxide [57]. This experiment was aimed to assess whether *A. hydaspica* prevented oxidative damages to erythrocyte membrane or not. *Acacia hydaspica* methanol extract and its various fractions showed differential pattern of anti-hemolytic activity. Results indicated that AHM, AHE and AHB exhibited potent anti-hemolytic action in a dose dependent way. However maximum inhibition of hemolysis was exhibited by AHE (97 % RBC membrane stabilization) at 1000 μg/ml, whereas aqueous fraction exhibit minimum anti-hemolytic activity as compared to other fractions (Table 4). Lysis of erythrocytes was displayed to be decreased with an increase in concentration of extract or fraction.

**Table 4:** Anti-hemolytic activity of *A. hydaspica* methanol extract and its soluble fractions against H_2_O_2_ induced hemolysis

Samples	Positive control (A)	Negative control (B)	Optical density^C^ at 560 nm concentration (μg/ml)				% Inhibition of hemolysis at 1000 (μg/ml)
			100	250	500	1000	
AHM	1.45 ± 0.02	0.11 ± 0.05	0.69 ± 0.01	0.85 ± 0.05	0.96 ± 0.05	1.15 ± 0.01	79.3^c^
AHH	1.45 ± 0.03	0.11 ± 0.05	0.65 ± 0.05	0.71 ± 0.05	0.88 ± 0.05	0.98 ± 0.05	67.6^d^
AHC	1.45 ± 0.05	0.11 ± 0.05	0.51 ± 0.05	0.63 ± 0.03	0.71 ± 0.05	0.87 ± 0.02	60.0^e^
AHE	1.45 ± 0.06	0.11 ± 0.05	0.72 ± 0.04	0.89 ± 0.09	1.14 ± 0.01	1.42 ± 0.02	97.9^a^
AHB	1.45 ± 0.04	0.11 ± 0.05	0.67 ± 0.05	0.81 ± 0.05	0.92 ± 0.05	1.29 ± 0.03	88.9^b^
AHA	1.45 ± 0.07	0.11 ± 0.05	0.49 ± 0.05	0.59 ± 0.05	0.64 ± 0.05	0.79 ± 0.05	54.5^f^
QR	1.45 ± 0.08	0.11 ± 0.05	0.72 ± 0.04	0.89 ± 0.09	1.14 ± 0.01	1.43 ± 0.02	98.6^a^

Each value is represented as mean ± SEM (*n* = 3). Values in same column followed by different letter (^a–f^) are significantly different (*p* <0.05), A; phosphate buffer saline replaced extract/fraction as this treatment results in 0 % hemolysis, B; hydrogen peroxide replaced extract to serve as a negative control since this treatment results in 100 % hemolysis, ^c^; lower values are associated with increased cell lysis. *AHM A. hydaspica* methanol extract, *AHH A. hydaspica n*hexane fraction, *AHE A. hydaspica* ethyl-acetate. (Completely Randomized AOV followed by Tukey HSD All-Pairwise Comparison Test)

The outcome of the current experiment presents the occurrence of primary antioxidants, which possess anti-hemolytic effect.

### Assessment of anticancer potential by MTT assay

Maximum numbers of the anticancer drugs presently consumed in chemotherapy are cytotoxic to healthy cells, consequences in excessive anomalies. Based on the antioxidant ability of the extract/fractions of *A. hydaspica*; AHM, AHE and AHB were selected for cytotoxic testing against cancer and normal cell lines. The integration of cancer and normal cells in the study design are necessary for the detection of cyto-selective compounds. The result of MTT assay revealed that tested samples inhibited cell proliferation in a dose dependent manner, although their effects were different from one another. IC_50_ values of extract/fractions at all tested cell lines exhibited a distinctive pattern (Table 5). AHM, AHE and AHB showed no cytotoxicity against normal Vero cells as shown by higher IC_50_ values (IC_50_ > 250 μg/ml). AHE was found the most effective against cancer cells, with IC_50_ values found to be 29.9 ± 0.909 μg/ml for MDA-MB-361 cell line and 39.5 ± 0.872 μg/ml for HCC-38 cell line. IC_50_ values and selectivity indices (SI) of different samples against cancer cells and normal cells were shown in Table 5. Most of the anticancer drugs currently used in chemotherapy are cytotoxic to normal cells, leading to unwanted side effects. The current study provides a significant bearing in search for compounds, which can reduce the harmful side effects of anticancer drugs, as *A. hydaspica* extract/fractions showed significant cyto-selective effect indicated by higher selectivity index values. Phytochemical investigation indicates the presence of bioactive metabolites in active extract/fractions might contribute to inhibiting cell viability in cancer cells. Our results are in agreement with the previous study of Kalaivani et al., demonstrating that distinct effect of *A. nilotica* extracts in each cell line might be due to the phyto-diversity or varied mechanisms accompanying each of the compounds [58]. Flavonoids have been presented little or no cytotoxic effect on healthy cells while being cytotoxic against various human cancer cells [59]. Flavonoids mediate their actions by various ways i.e., simply binds to the cell membrane, penetrate in vitro cultured cells or via modulation of the cellular metabolic activities. Extenuation of oxidative damage, carcinogen inactivation, inhibition of cell growth and differentiation, induction of cell cycle arrest and apoptosis, diminishing of tumor angiogenesis and restriction of metastasis are the major implications of flavonoids anti-carcinogenic activities [60, 61]. The cyto-protective effect of extract against normal cell depict that *A. hydaspica* selectively inhibited the growth of cancer cell types and did not induce cell death in normal cells indicating the anticancer activity of *A. hydaspica.* This calls for further studies on the active components for proper assessment of their chemotherapeutic properties as well as their possible development as promising anticancer drugs.

**Table 5:** Cytotoxic effect of *A. hydaspica* methanol extract and its derived fractions on MDA-MB 361, HCC-38 and Vero cells of green monkey after 48 h of treatment

Extract/Fraction	MDA361 cell line	SI	HCC38 cell line	SI	Vero cell line
	IC_50_ (μg/ml)	IC_50_V/IC_50_M	IC_50_ (μg/ml)	IC_50_V/IC_50_H	IC_50_ (μg/ml)
AHM	46.9 ± 1.31*	5.42	75.9 ± 1.32*	3.34	254 ± 1.81
AHE	29.9 ± 0.91*	9.83	39.5 ± 0.87*	7.44	294 ± 1.55
AHB	37.1 ± 1.01*	6.97	56.1 ± 0.93*	4.60	258 ± 1.68

Each value expressed as mean ± SEM (*n* = 3). Selectivity index (SI) >3 is considered to be highly selective, * shows significance at *p* <0.001 as compared to Vero cells. M represents MDA-MB-361; V represents Vero and H represents HCC38 cell lines. (One way ANOVA followed by Tukey’s multiple comparison test)

### HPLC-DAD analysis

**Fig. 2:**
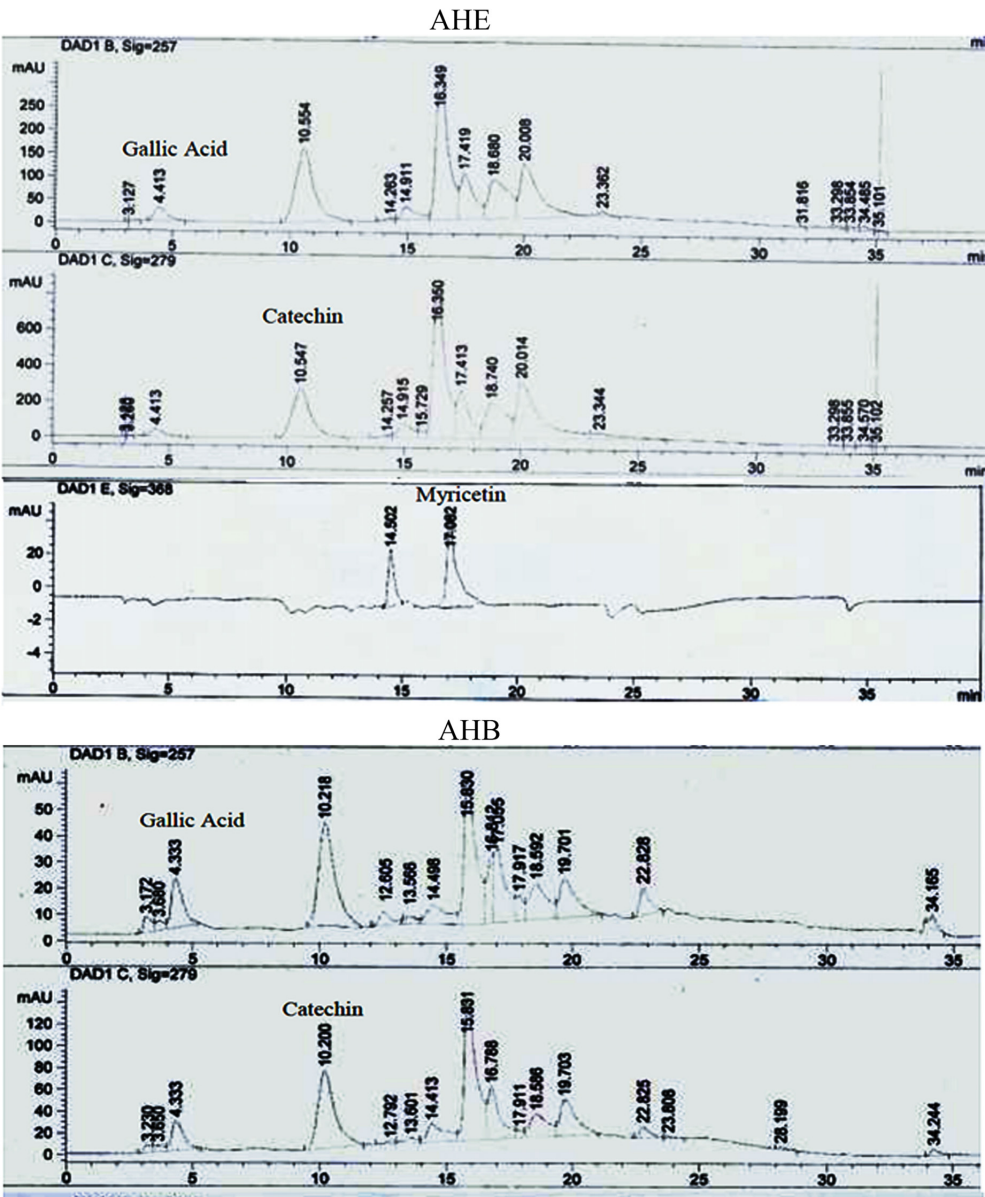
HPLC chromatogram of AHE and AHB fractions of *A. hydaspica*

HPLC-DAD analysis is the best way for chemical profiling of plant extract, therefor rapid, reproducible and specific finger printing was established in the current research work. AHB and AHE showed excellent antioxidant, antihemolytic and anticancer activities hence subjected for chemical profiling by HPLC-DAD. Gallic acid, catechins, caffeic acid, rutin, kaempferol, myricetin and Our result correlate well with quercetin were used as markers to compare retention time and UV absorbance with test samples. Table 6 indicated the retention time, optimized signal wavelength, and regression analysis of reference flavonoids. HPLC-DAD chromatogram reveals the presence of two known compounds gallic acid (144.70 μg/100 mg dry powder) and catechin (3995.208 μg/100 mg dry powder) in AHB, whereas AHE showed three reference compounds with maximum amount shown by catechin (8648 μg/100 mg dry powder) followed by gallic acid (52.92 μg/100 mg dry powder) and myricetin (34.60 μg/100 mg dry powder) (Fig. 2a and b, Table 7).

**Table 6:** Retention time, optimized signal wavelength, and regression analysis of reference flavonoids determined by HPLC-DAD analysis

Compound	Signal wavelength	Retention time (min)	Regression analysis	R^2^
Gallic acid	257	4.35	y = 3.0565x + 8.01	0.9998
Rutin	257	15.91	y = 1.7773x−48.49	0.9958
Catechin	279	9.70	y = 1.1764x + 7.96	0.989
Caffeic acid	325	325	y = 12.569x + 22.66	0.9905
Apigenin	325	23.53	y = 12.950x−52.12	0.9957
Myricetin	368	18.72	y = 2.560x−15.09	0.9994
Kaempherol	368	23.59	y = 10.967x + 125.59	0.9964
Quercetin	368	21.31	y = 4.8624x + 35.01	0.9993

**Table 7:** HPLC-DAD profile of *A. hydaspica* ethyl acetate and n-butanol fractions

Extract/fraction	Compounds	Signal wavelength	Retention time	Quantity (μg/100 mg dry powder)
AHE	Gallic acid	275 nm	4.52	52.92
	Catechin	279 nm	11.43	8648.0
	Myricetin	368 nm	17.08	34.60
AHB	Gallic acid	257 nm	4.41	144.70
	Catechin	279 nm	10.55	3995.21

The standards were selected on the basis of their reported medicinal properties, for instance; catechin is an important phenolic compound with diverse beneficial health effects and its metabolites have shown therapeutic potential as antioxidant, anti-apoptotic, inhibit proliferation of breast cancer cells, block carcinogenesis and its effect is more pronounced in cancer cells as compared to normal cells [62]. Gallic acid (GA) possesses potent antitumoral and antioxidant properties. The research conducted by Ali et al. also illustrate that gallic acid and polyphenols in the acetone extract of *A. nilotica,* are responsible for cytotoxic activity [63]. Myricetin is also able to induce apoptosis of pancreatic cancer cells*,* human bladder carcinoma cell line, trigger apoptosis, regression of tumor growth, decrease metastasis and it increase bioavailability of tamoxifen, a drug used to treat breast cancer [64].

Our result correlate well with the research of Tung at al., reporting gallic acid, catechin, myricetin along with other polyphenols in ethyl acetate fraction of *A. confusa* leaves extract were responsible for the significant antioxidant and anticancer potential [53]. This calls for further studies on the active components for proper assessment of their chemotherapeutic properties as well as their possible development as promising anticancer drugs.

## Conclusion

The present study demonstrates the phytochemical profiling, in vitro antioxidant, anti-hemolytic and cyto-selective anticancer activity of *A. hydaspica* aerial parts extracts. Extracts with higher antioxidant capacity also had higher polyphenol content. It can be concluded that the extract obtained using higher polarity solvents were more effective radical scavengers then those obtained using less polar solvents. Ethyl acetate and *n*-butanol showed better characteristics as solvent for phenolic compounds. Furthermore these fractions tended to possess superior activity in lipid peroxidation inhibition and β-carotene bleaching assay as compared to BHA and BHT. Therefore, they might be used as preservative ingredients in the food and/or pharmaceutical industry. Moreover safety profile and chemotherapeutic potential of active fractions and methanol extract was determined by assessing the anti-hemolytic activity and in vitro testing against both cancer and normal cell lines. Bioactive compounds present in *A. hydaspica* active fractions might work synergistically and specifically in inhibiting proliferation of breast cancer cells with high SI value, suggesting that they might be used as a natural additive in human diets for cancer chemoprevention. However the evaluation and the discovery of new anticancer agents is long-term process that encompasses many steps by step approaches with the screening for anticancer properties, followed by the isolation and identification of bioactive compounds and finally in vivo anticancer activity testing in order to verify the aptitude of the compounds. Therefore further research would be required before such uses could be proposed with confidence.

## Abbreviations

ABTS, 2,2′-azino-bis (3-ethylbenzothiazoline-6-sulphonic acid; AHA, *Acacia hydaspica* residual aqueous fraction of methanol extract of aerial parts; AHB, *Acacia hydaspica* n-butanol fraction of methanol extract of aerial parts; AHC, *Acacia hydaspica* chloroform fraction of methanol extract of aerial parts; AHE, *Acacia hydaspica* ethyl acetate fraction of methanol extract of aerial parts; AHH, *Acacia hydaspica* n-hexane fraction of methanol extract of aerial parts; AHM, *Acacia hydaspica* methanol extract of aerial parts; BHA, butylated hydroxyanisole; BHT, butylated hydroxytoluene; DMEM/F12, Dulbecco’s modified eagle medium: nutrient mixture F-12; DPPH, 2,2-Diphenyl-1-Picrylhydrazyl; EDTA, ethylene diamine tetra acetic acid; PBS, phosphate buffer saline; PMS, phenazine methosulphate; TBA, thiobarbituric acid; TBARS, thiobarbituric acid reactive substances; TFC, total flavonoid content; TPC, total phenolic content
